# Effect of iron deficiency anemia on HbA1c in diabetic patients at Tikur Anbessa specialized teaching hospital, Addis Ababa Ethiopia

**DOI:** 10.1186/s12878-018-0132-1

**Published:** 2019-01-09

**Authors:** Absra Solomon, Mintewab Hussein, Mikias Negash, Abdurezak Ahmed, Fitsum Bekele, Daniel Kahase

**Affiliations:** 1 College of Medicine and Health Sciences Department of Medical Laboratory Sciences, Wolkite University, Wolkite, Ethiopia; 2 College of Health Sciences, School of Allied Health Sciences, Department of Medical Laboratory Sciences, Addis Ababa University, Addis Ababa, Ethiopia; 3 College of Health Sciences, Department of Internal Medicine, Addis Ababa University, Addis Ababa, Ethiopia

**Keywords:** HbA1c, Iron deficiency anemia, Diabetic mellitus

## Abstract

**Background:**

Hemoglobin A1C (HbA1c) is the predominant hemoglobin found in HbA1 fractions. A1c assay is the recommended assay for diagnosing diabetes and any condition that changes red cell turnover such as Iron deficiency Anemia (IDA), will lead to spurious A1C results. Therefore, the present study was aimed at determining the effect of IDA on HbA1c in diabetic patients attending Black Lion Specialized Teaching Hospital, Addis Ababa, Ethiopia.

**Methods:**

A facility based comparative cross sectional study was conducted on 174 diabetic patients (87 with IDA and 87 without IDA) from April to July 2016. Socio demographic data and clinical conditions were collected using structured questionnaire. Venous blood was collected for performing Complete blood count (CBC) using Cell dyn 1800 hematology analyzer; Serum ferritin, performed by COBAS INTEGRA 400/800 Chemistry analyzer and HbA1c tests, performed by COBAS C 111 analyzer. Data was analyzed using SPSS version 21 software. Pearson’s correlation, chi-square, and independent t-tests were calculated. The data was presented as mean ± SD. A *P*-value of < 0.05 was taken as statistically significant.

**Results:**

Mean hemoglobin (Hgb), hematocrit (HCT), Mean cell volume (MCV), mean cell hemoglobin (MCH), mean cell hemoglobin concentration (MCHC) were lower in IDA group compared to non-IDA diabetic patients. HbA1c (%) level was significantly lower in IDA group (6.18 ± 1.57) compared with the non-IDA diabetic patients (7.74 ± 1.81) (*p* < 0.05).

**Conclusion:**

HbA1c is significantly lower in diabetic patients with IDA compared to the non-IDA diabetic patients. Therefore, the authors believe that monitoring these patients using only HbA1c could be misleading, hence physicians and health care providers should take this into account before making any therapeutic decision. Detailed examination including large number of participants employing advanced laboratory techniques is recommended.

**Electronic supplementary material:**

The online version of this article (10.1186/s12878-018-0132-1) contains supplementary material, which is available to authorized users.

## Background

Hemoglobin A1C (HbA1c) is the predominant hemoglobin found in HbA1 fractions and it constitutes 5% of the total hemoglobin in normal adults and up to 15% in patients with diabetes mellitus [1].

Hgb A to HbA1c conversion takes place during the entire life span of the red blood cell and the rate of this reaction is faster in diabetics because of the higher prevailing glucose concentration, resulting in a higher concentration of HbA1c [2].

Red blood cells (RBC) are freely permeable to the plasma glucose molecules, and hemoglobin is practically exposed to the same glucose concentrations as plasma [3]. Therefore, HbA1c level is directly proportional to average blood glucose concentration over the previous 4 weeks to 3 months or the average lifespan of the erythrocyte [4].

There are a number of methods available to estimate glycated hemoglobin like immunoturbidimetry, ion exchange high-performance liquid chromatography (HPLC), boronate affinity, and enzymatic method [5]. HbA1c level of ≥6.5% is sufficiently sensitive and specific to identify individuals who are at risk for developing retinopathy and who should be diagnosed as diabetic [6].

HbA1c assay is as an accurate and precise measure of chronic glycemic levels as it correlates well with the risk of diabetes complications for the same reason it is recommended to rely on HbA1c for diagnosing diabetes [7].

Despite its benefit, HbA1c is affected by a variety of genetic, physiological, hematological and illness-related factors [8]. Falsely elevated HbA1c concentrations are encountered when there is increased circulating erythrocyte life span (decreased red cell clearance) or impaired reticulocyte production. On the other hand, falsely decreased HbA1c level is seen in conditions with a reduced erythrocyte life span (increased hemoglobin turnover) or where a large number of reticulocytes are produced [8].

IDA, a diminished red cell production due to low iron stores in the body, is the most nutritional disorder worldwide and accounts for half of anemia. IDA can result from inadequate iron intake, decreased iron absorption, increased iron demand and increased iron loss [9]. Patients with diabetes may become more vulnerable to the effects of anemia. The definitive test for diagnosis of IDA is bone marrow aspiration .however as the procedure is invasive, difficult and expensive; serum ferritin is found to be the alternative test for distinguishing those with IDA from those who are not iron deficient [10].

Globally, anemia affects 1.62 billion people which correspond to 24.8% of the world population [11].IDA in the general population is a common cause of anemia and is prevalent in patients with DM [12].

Anemia is under recognized and largely untreated in patients with diabetes, in Ethiopia .to the best of our knowledge there is no study conducted in Ethiopia on the effect of IDA on HbA1c in diabetic patients. Hence, we have planned to investigate the effect of IDA on HbA1c in diabetic patients attending black lion Specialized teaching Hospital in Addis Ababa, Ethiopia.

## Methods

### Study area and study period

Tikur Anbessa Specialized Teaching Hospital is the largest teaching hospital in Ethiopia. It offers diagnosis and treatment for approximately 370,000–400,000 patients a year. The hospital has different units and clinics that provide specialized service for patients. The diabetes clinic is among the specialized units in the hospital which offers services for about 70–90 diabetic patients per day. The study was conducted from April to July 2016.

### Study design

Facility based Comparative crossectional study design was implemented.

### Source population

All diabetic patients who visited Tikur Anbessa Specialized Teaching Hospital.

### Study population

All diabetic patients with IDA and baseline laboratory data and who were attending Black Lion Specialized Teaching Hospital Diabetic clinic during the study period.

### Questionnaire and clinical examination

Socio-demographic characteristics were collected using structured questionnaires. An Enrollment form containing past medical history pertaining to chronic diseases such as kidney disease, heart related problem, skin diseases, blood coagulation disorders and other medical complaints were completed for every individual. All patients were asked to provide a detailed history and were subjected to a physical examination. The levels of hemoglobin, MCH, hematocrit, MCV, MCHC, platelet count, total leucocyte count (TLC), and differential leucocyte count (DLC) were measured.

### Questionnaire validation

A one-day orientation was given for both the data collectors and the supervisors to explain the objectives of the study, the contents of the questionnaire, issues related to the confidentiality of the responses, the rights of respondents and the data collection processes.

To ensure the clarity and validity of the instruments, the questionnaires were pretested on 5 % of the sample size in Black lion teaching hospital before the study was undertaken by the actual data collectors. After the pre-test, all the participants were directly contacted and asked about the clarity questions in the instrument. Besides, the data collectors were also asked if there was any kind of difficulty on the data collection process. Accordingly, some modifications were made on the instrument before the actual data collection process Additional file 1.

### Specimen collection and laboratory processing

All diabetic patients with and without IDA data who visited Black Lion Specialized Teaching Hospital during the study period and fulfilling the inclusion criteria were included.

Five mili liter of venous blood was collected by needle and syringe technique aseptically from each of the study participants. CBC, serum ferritin and HbA1c tests were done following manufacturers procedure for running each test. CBC was done by using Cell dyn 1800 hematology analyzer, HbA1c by COBAS C 111 analyzer and Serum ferritin was determined using COBAS INTEGRA 400/800 Chemistry analyzer. On the basis of hemoglobin levels, IDA patients were categorized as having mild, moderate, or severe anemia [10].

### Data analysis

Data was entered into Microsoft Excel, exported to SPSS version 21 and analyzed by the same. Frequency and summary statistics were used to describe the distribution of age, sex among the IDA and the control group. Pearson’s Chi-square test was used to determine the association between hematological parameters and HbA1c.Independent t-test was calculated for comparison of the hematological parameters and HbA1c mean between the IDA and non-IDA Diabetic patients. A *P*-value of < 0.05 was taken as statistical significant.

## Results

### Socio demographic characteristics

A total of 174 diabetic patients (87 with IDA and 87 without IDA) participated in the study. Out of 174 diabetic patients, 89(51.1%) were male and 85 (48.9%) were female. The mean age was 47.5 ± 15.83 (Table 1).

**Table 1:** Age group and sex distribution among the study population at Tikur Anbessa Specialized Teaching Hospital from April to July 2016

Variable	IDA Group #(%)	Non-IDA Group #(%)	Total
Sex			
M	53(60.9)	36(41.4)	89
F	34(39.1)	51(58.6)	85
Age Group			
18–27	12(13.8)	9(10.3)	21
28–37	21(24.2)	11(12.7)	32
38–47	13(14.9)	17(19.5)	30
48–57	11(12.7)	21(24.2)	32
58–67	20(22.9)	19(21.8)	39
+ 68	10(11.5)	10(11.5)	20
Total	87	87	174

A total of 87 patients diagnosed with IDA were involved in the study, where 53 (60.9%) were male and 34 (39.1%) were female. Of the 87 non-IDA diabetic patients, 51 (58.6%) were female and 36 (41.4%) were male.

### Comparison of HbA1c and hematological parameters among IDA and non-IDA group

All hematological parameters, serum ferritin and HbA1c were examined for both groups. Mean ± SD was calculated and Independent t-test was used to compare the mean of RBC, Hgb, HCT, MCV, MCH, MCHC, HbA1c among IDA and non- IDA groups. The mean RBC, Hgb, HCT, MCV, MCH, MCHC, HbA1c were significantly lower in IDA group compared to the control group (*P* < 0.05) (Table 2).

**Table 2:** Independent t test for hematological parameters among the IDA and Non-IDA groups at Tikur Anbessa Specialized Teaching Hospital from April to July 2016

Parameters	IDA	Non-IDA	t-test 95% CI		
			*P*-value	Lower	Upper
RBC	3.45 ± 0.80	4.91 ± 0.39	.000	− 1.65032	− 1.26945
Hgb	9.97 ± 2.04	15.17 ± 1.21	.000	− 5.7010	−4.6944
HCT	30.43 ± 6.37	45.29 ± 3.53	.000	− 16.4000	−13.3126
MCV	88.5 ± 8.56	92.12 ± 3.76	.001	−5.5368	−1.5759
MCH	29.89 ± 4.04	30.93 ± 1.62	.027	−1.9672	−.1224
MCHC	32.97 ± 2.19	33.57 ± 0.99	.022	−1.1073	−.0881
RDW	19.08 ± 4.24	14.42 ± 1.66	.000	3.7013	5.6297
HbA1c	6.18 ± 1.57	7.74 ± 1.81	.000	−2.05946	−1.04169

### Association between RBC, red cell indices and HbA1C

The mean RBC, MCV, MCH, MCHC, RDW were 3.45 ± 0.8, 88.57 ± 8.56, 29.89 ± 4.04, 32.97 ± 2.19, 3.45 ± 0.80 respectively. Pearson correlation test was used to determine the association between HbA1C and hematological parameters of the IDA patients. HbA1C was statistically non-significant with RBC, MCV, MCH, MCHC (*p* > 0.05) (Table 3).

**Table 3:** Association between RBC, red cell indices and HbA1C at TASH from April to July, 2016

Parameters	Mean	HbA1C	
		Pearson correlation r value	*P* value
RBC	3.45 ± 0.8	−0.10	0.31
MCV	88.57 ± 8.56	− 0.28	0.79
MCH	29.89 ± 4.04	− 0.08	0.41
MCHC	32.97 ± 2.19	−0.73	0.49
RDW	19.08 ± 4.24	−0.10	0.35

### Severity of anemia in IDA diabetic patients

**Fig. 1:**
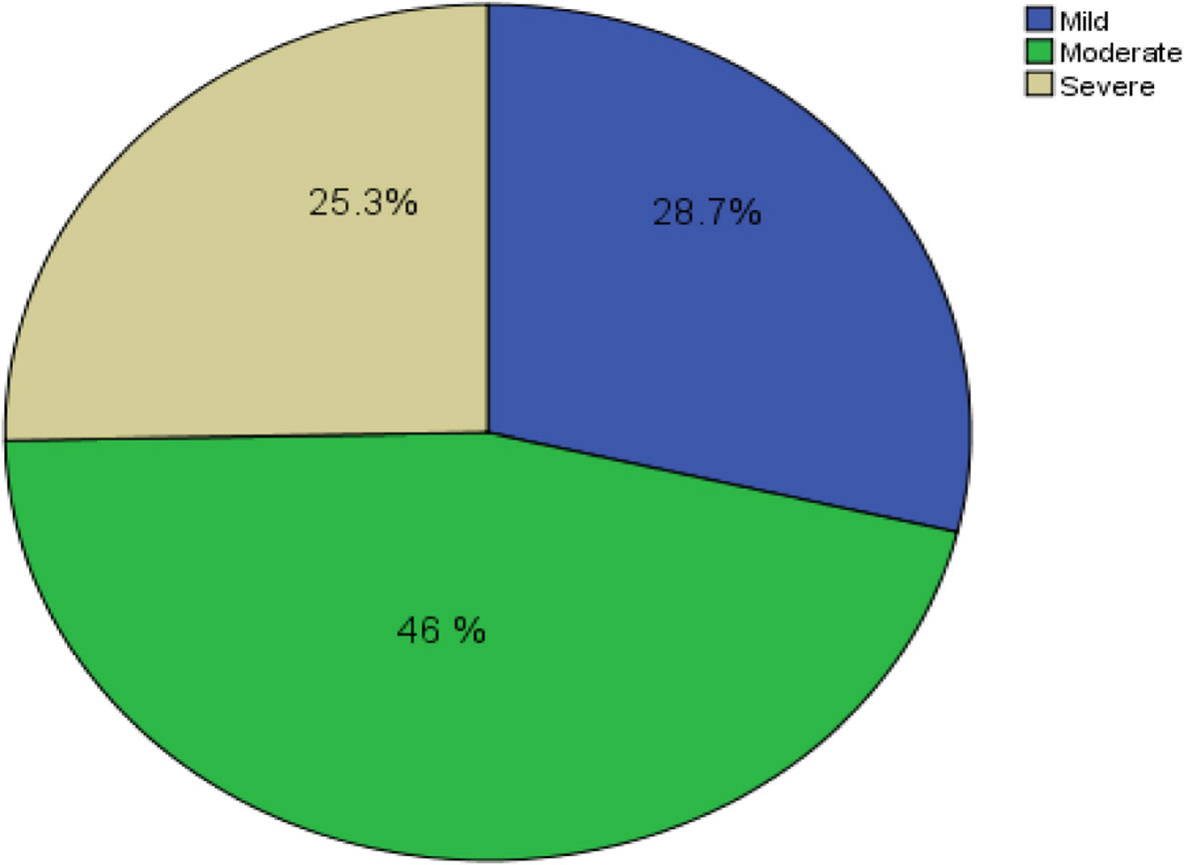
Pie chart of frequency of severity of anemia among the IDA diabetic patient at Tikur Anbessa Specialized Teaching Hospital from April to July 2016

Frequency was calculated to determine the degree of anemia among the 87 IDA diabetic patients. Mild anemia was seen in 25 (28.7%) patients, moderate anemia in 40 (46%) patients and severe anemia was seen in 22 (25.3%) of the patient (Fig. 1).

## Discussion

HbA1c has emerged as a marker of glycemic control, glycemic risk and predictor of diabetic complication and as screening tool for diagnosis of DM [7]. Anemia may either increase or reduce the HbA1c values due to changes in the half-life of RBC [13]. Numerous studies have been conducted on the effect of iron deficiency anemia on HbA1c in diabetic patient or non-diabetic patients and different results have been obtained but there is no clear explanation on mechanism how iron-deficiency affects HbA1c.

The present study revealed that HbA1c (%) is significantly lower in IDA group (6.18 ± 1.57) compared to the control group (7.74 ± 1.81) (*p* < 0.05). This is supported by studies done by Sinha et al.*,* in 2012 [3], Cavagnolli *et a., l* in 2015 [12] and Kalasker et al.*,* in 2014 [13]. They all stated that HbA1c concentration tends to be lower in the presence of iron deficiency anemia. According to Sinha et al.*,* suggestion, the reason for lower HbA1c is due to the severity of anemia in the study participants. In contrary, Ford et al.*,* in 2011 [14], Silva et al.*,* in 2015 [15], Shekhae et al.*,* in 2014 [16] and Chhabra et al.*,* in 2015 [17] have obtained higher HbA1c level in IDA patients.

Association between RBC, red cell indices and HbA1c were determined in IDA group and the result was not statistically significant. Similarly, a study done in India in 2014 [18] showed no significant correlation, but a borderline significant association was observed between MCH and HbA1c in IDA diabetic patient (*P* = 0.05). Among the hematological parameters; RBC, Hgb, MCV, MCH showed statistically significant mean difference between the two groups. This finding is similar with a study done in India in 2016 [19].

On the basis of hemoglobin, iron deficient patients fall under three groups: mild anemia, moderate anemia and severe anemia [13]. Based on this classification, 25 (28.7%) of patients had mild, 40 (46%) moderate and 22 (25.3%) severe anemia. In a similar Study done in India, Severe anemia was seen in 38 (76%) patients, and moderate in 12 (24%) patients [15]. The present study revealed no significant association between Sex, Age and HbA1c in IDA diabetic patients, which is in agreement with similar study done in India [18].

## Conclusion

In general this study has showed that, patients with IDA have significantly lower HbA1c compared to non-IDA diabetic patients. Monitoring these patients using HbA1c could be misleading because their actual HbA1c level could be lower than the actual value. Hence, physicians and health care providers should take this into account before making any therapeutic decision. They should also consider treating the iron deficiency anemia before diagnosing the diabetes using HbA1c. Detailed examination including large number of participants employing advanced laboratory techniques is recommended.

## Additional file

Additional file 1: Questionnaire Effect of Iron Deficiency Anemia on HbA1c in Diabetic Patients at Tikur Anbessa Specialized Teaching Hospital, Addis Ababa Ethiopia April 2016. (DOCX 30 kb)

## Abbreviations

CBC: Complete blood count
HbA1c: Hemoglobin A1C
HCT: Hematocrit
Hgb: Hemoglobin
HPLC: High-performance liquid chromatography
IDA: Iron deficiency Anemia
MCH: Mean cell hemoglobin
MCHC: Mean cell hemoglobin concentration
MCV: Mean cell volume
RBC: Red blood cells

## References

[1] Smith RJ, Koenig RJ, Binnerts A, Soeldner JS, Aoki TT (1982). Regulation of hemoglobin AIC formation in human erythrocytes in vitro: effects of physiologic factors other than glucose. J Clin Invest.

[2] Nanji AA, Pudek MR (1983). Glycosylated hemoglobin: a review. Can Fam Physician.

[3] Sinha N, Mishra TK, Singh T, Gupta N (2012). Effect of Iron deficiency Anemia on hemoglobin A1c levels. Ann Lab Med.

[4] Kim S, Min WK, Chun S, Lee W, Park HI (2011). Glycated albumin may be a possible alternative to hemoglobin A1Cin diabetic patients with anemia. Clin Chem Lab Med.

[5] Singh K, Rao J, Mahdi JJ (2014). Standard recommendation for interpretation of HbA1c graph in ion exchange HPLC method. Int J Med Invest.

[6] Mahajan RD, Mishra B (2011). Using glycated hemoglobin HbA1c for diagnosis of diabetes mellitus: an Indian perspective. Int J Biol Med Res.

[7] American Diabetes Association (2010). Diagnosis and classification of diabetes mellitus. Diabetes Care.

[8] Gallagher EJ, Roith DL, Bloomgarden Z (2009). Review of hemoglobin A1c in the management of diabetes. J Diabete.

[9] Short MW, Domagalski JE (2013). Iron deficiency anemia: evaluation and management. Am Fam Physician.

[10] World Health Organization (2011). Hemoglobin concentrations for the diagnosis of anemia and assessment of severity. Vitamin and mineral nutrition information system.

[11] World Health Organization (2015). The global prevalence of anemia in 2011.

[12] Cavagnolli G, Pimentel AL, Freitas PAC, Gross JL, Camargo JL (2015). Factors affecting A1C in non-diabetic individuals: review and meta-analysis. Clin Chim Acta.

[13] Kalasker V, Madhuri S, Kodliwadmath MV, Bhat H (2014). Effect of Iron deficiency Anemia on glycosylated hemoglobin levels in non-diabetic Indian adults. Int J Med Health Sci.

[14] Ford ES, Cowie CC, Li C, Handelsman Y, Bloomgarden ZT (2011). Iron-deficiency anemia, non-iron-deficiency anemia and HbA1c among adults in the US. J Diabete..

[15] Siliva JF, Pimentel AL Silva JF, Camargo JL. Effect of iron deficiency anemia on HbA1c levels is dependent on the degree of anemia. Clin.Biochem. 2015. 10.1016/j.clinbiochem.2015.09.004. 10.1016/j.clinbiochem.2015.09.004 26365695

[16] Shekhar H, Mangukiya KK, Kaur A, Jadeja P (2014). Effect of Iron deficiency on glycation of hemoglobin in non-diabetics. IJSN.

[17] Chhabra RJ, Dhadhal R, Sodvadiya K (2015). Study of glycated Haemoglobin (HbA1c) level in non-diabetic Iron deficiency Anemia. IJIRR.

[18] Christy AL, Manjrekar PA, Babu RP, Hegde A, Rukmini MS (2014). Influence of Iron deficiency Anemia on hemoglobin A1C levels in diabetic individuals with controlled plasma glucose levels. Iranian Biomed.

[19] Manisha G, Nitin S, Ranjana M, Singh GA, Ritu G (2016). Study of glycosylated hemoglobin in Iron deficiency Anemia. Sch J App Med Sci.

