# Supplementary material for: Effect of iron deficiency anemia on HbA1c in diabetic patients at Tikur Anbessa specialized teaching hospital, Addis Ababa Ethiopia
Source: BMC Hematol. 2019 Jan 9;19:2. doi: 10.1186/s12878-018-0132-1 (PMC6327502; doi:10.1186/s12878-018-0132-1)
Supplement: Supplementary file 1 — Questionnaire Effect of Iron Deficiency Anemia on HbA1c in Diabetic Patients at Tikur Anbessa Specialized Teaching Hospital, Addis Ababa Ethiopia April 2016. (DOCX 30 kb) [file 12878_2018_132_MOESM1_ESM.docx]

## Effect of Iron Deficiency Anemia on HbA1c in Diabetic Patients at Black Lion Specialized Teaching Hospital, Addis Ababa Ethiopia

Part one : General information

1. Patient code ___________
2. Gender
3. Male B. Female
4. Age
5. 18-27
6. 28-37
7. 38-47
8. 48-57
9. 58-67
10. ≥68

Part two: Medical record

1. Body temperature __________
2. Weight ___________
3. Blood pressure_________
4. Plasma glucose concentration____________
5. HCG (pregnancy)____________

Part three: Previous History

1. Do you have a renal failure history?
2. Yes B. No
3. Did you have any recurrent malaria infection in the past 3 months?
4. Yes B. No
5. Do you have a history of blood loss in the past 3 months?
6. Yes B. No
7. Have you donated blood in the past 3 months?
8. Yes B. No
9. Have you taken a history of iron treatment?
10. Yes B.
